# The old and the large may suffer disproportionately during episodes of high temperature: evidence from a keystone zooplankton species

**DOI:** 10.1093/conphys/coaa038

**Published:** 2020-09-08

**Authors:** Tim Burton, Sigurd Einum

**Affiliations:** Centre for Biodiversity Dynamics, Department of Biology, Norwegian University of Science and Technology, Realfagbygget, NO-7491 Trondheim, Norway

**Keywords:** body size, Daphnia, knock-down time, temperature sensitivity, thermal death curves

## Abstract

Widespread declines in the body size of aquatic ectotherms have been attributed to the poorer ability of older, larger individuals to tolerate high temperature. Here, using the thermal death time curve framework, we investigate the relationship between temperature tolerance and size/age by measuring the change in heat tolerance of the keystone zooplankton species *Daphnia magna* across a range of temperature intensities (and hence exposures of varying duration) among individuals that differed up to 3-fold in size and thus varied in age also. Across the gradient of exposure temperatures, younger, smaller individuals were more tolerant than older, larger individuals. This suggests that the young and the small may be better equipped to withstand temperature challenges that are both intense/brief and more moderate/prolonged. Our study generalizes results obtained from more acute tolerance assays, providing physiological evidence consistent with the observed reductions in ectotherm body size as a response to warming in aquatic systems.

## Introduction

**Figure 1:**
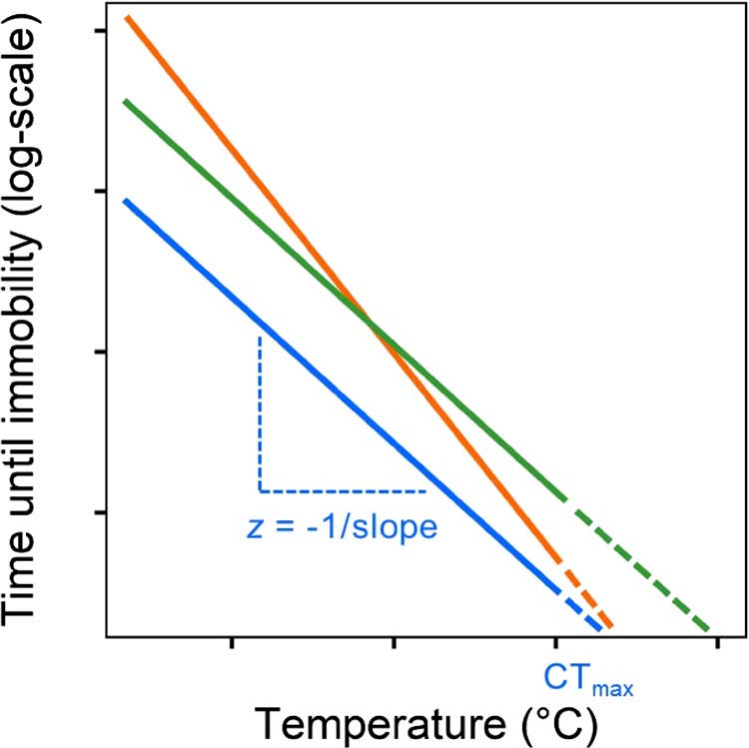
Hypothetical thermal death time curves (TDTCs). A given TDTC may represent e.g. different species, populations or treatment groups and is quantified by a linear regression of knock-down time (*t*, on log scale) on exposure temperature (*T*). The temperature sensitivity coefficient *z* (−1/slope) obtained from this regression describes how heat tolerance changes with exposure temperature. Perhaps counterintuitively, a high value of *z* reflects a low sensitivity, but we choose to follow this definition in the current paper for consistency with previous literature (Rezende *et al.*, 2014; Jørgensen *et al.*, 2019). The elevation of this relationship represents the overall magnitude of heat tolerance across temperatures and the *x*-intercept, CTmax (the temperature resulting in physiological failure when log *t* = 0, obtained by extrapolating the regression until it intersects the *x*-axis, shown here by the dashed lines). Where groups have similar temperature sensitivities (e.g. blue vs. green lines), the main difference is in the elevation of the relationship, representing the overall magnitude of heat tolerance across temperatures. Accordingly, the group represented by the green line is always more heat-tolerant than the group represented by the blue line and the proportional difference in knockdown time between the two is equal across temperatures. Where the temperature sensitivity *z* varies (e.g. blue vs. orange lines), relative heat tolerance depends on exposure temperature. Thus, the group represented by the orange line is most tolerant of the mildest exposure, but its tolerance declines proportionately more with increasing temperature than that of the blue group.

Size is perhaps the most ubiquitous trait measured in organismal biology, being integrally related to variation in life history and physiology (Kleiber, 1947; Martin *et al.*, 1993; Calder, 1996). Evidence continues to accumulate for a decline in body size of aquatic organisms as environments warm (Daufresne *et al.*, 2009; Forster *et al.*, 2012; Evans *et al.*, 2019), with such ‘shrinking’ being proposed as a third universal response to global environmental change (Gardner *et al.*, 2011). Seeking an explanation for this phenomenon, the negative covariation between acute heat tolerance and body size (and thus also age due to the confound between the two traits, see Bowler *et al.*, 2008) has provided empirical support (Brans *et al.*, 2017; Clark *et al.*, 2017; Di Santo *et al.*, 2017; Messmer *et al.*, 2017; Burton *et al.*, 2018). Notably though, some studies do not conform to this trend. In a comparison of seven reef fish species, only three out of the seven species tested showed relationships between CTmax and body size. Furthermore, these relationships were either weakly negative or even positive (Ospina *et al.*, 2004). In a separate comparison of several North American salmonids and sport fishes, only two out of the six species tested demonstrated a negative relationship between size and CTmax (Recsetar *et al.*, 2012). The reasons for these discrepancies are unclear. On the one hand, they may reflect genuine biological variation among species. However, it is often not apparent if the size of the individuals tested approximates the typical range achieved by the species in nature. Moreover, differences in the manner in which standard tolerance assays are implemented may also have contributed to inconsistency in the reported relationships between acute heat tolerance and size/age (Terblanche *et al.*, 2007; Chown *et al.*, 2009). Acute heat tolerance is most frequently measured in units of temperature at which locomotory function ceases during continuous ‘ramping’, or time (to loss of locomotory function at a single constant temperature, Lutterschmidt *et al.*, 1997a, Lutterschmidt *et al.*, 1997b, Terblanche *et al.*, 2011). However, like any other stressor, the physiological challenge imposed by high temperature depends on both these variables, i.e. its intensity *and* for how long it is experienced (Bigelow, 1921). We propose that our understanding of how heat tolerance varies across the lifespan has been hampered by implementation of standard tolerance assays that quantify only a single dimension (i.e. temperature or time) of a response that is better described by an interaction between the two (Rezende *et al.*, 2014). The cladoceran component of zooplankton communities, particularly members of the genus *Daphnia*, are an integral part of aquatic systems, as they are effective filter feeders on phytoplankton and are a key food source for planktivorous organisms. *Daphnia* are well suited to investigating the relationship between temperature tolerance and size/age because they develop directly after hatching, meaning that differences in size are not confounded with major developmental transitions in morphology/life stage (with the exception of sexual maturation which is evident in *Daphnia* when eggs become visible within the carapace). *Daphnia* are also able to reproduce clonally, meaning that measurements of temperature tolerance can be performed in a manner that is free of variation attributable to genetic differences among individuals. Here, using the species *Daphnia magna* as a model organism, we employ ‘thermal death time curves’ (hereafter, TDTCs) to investigate how heat tolerance varies in relation to size (and thus also age) as a function of both the intensity and duration of thermal stress (Rezende *et al.*, 2014).TDTCs draw upon the well-founded negative exponential relationship between the intensity of heat stress and the time over which it can be tolerated (Bigelow, 1921) by using multiple static temperature exposures to describe physiological tolerance (in units of time) across a range of temperatures (Rezende *et al.*, 2014). As such, TDTC’s offer a standardizable framework for measuring heat tolerance and can be described as

$$\begin{align*} \log 10\ t=\frac{\left(\mathrm{CTmax}-T\right)}{z} \end{align*}$$

where *T* corresponds to the exposure temperature (°C), *t* the time until ‘knock-down’ (min), CTmax the upper thermal limit (the temperature resulting in physiological failure when log *t* = 0), and *z* a constant that describes the sensitivity to temperature change (Fig. 1). The temperature sensitivity coefficient *z* can be obtained from a semi-logarithmic regression of ‘knock-down’ time on exposure temperature (−1/slope from this regression) and describes how heat tolerance changes with exposure temperature. As such, TDTCs have the capacity to reveal patterns with the potential to explain inconsistencies in the reported relationship between heat tolerance and size/age as obtained through standard (i.e. relatively acute) tolerance assays. For example, if *z* varies with respect to size/age it might suggest the presence of adaptations or mechanisms that result in younger or smaller individuals having greater tolerance of mild, prolonged temperature exposures, but perhaps similar tolerance of intense, brief episodes of high temperature (e.g. comparison of orange vs blue lines in Fig. 1). Here, we focus on both the temperature sensitivity coefficient, *z*, and the elevation of the TDTC, which represents the overall magnitude of heat tolerance across exposure temperatures to test the prediction that heat tolerance declines throughout life in aquatic ectotherms.

## Materials and methods

Ten cultures of a single genotype of *Daphnia magna* were maintained in 1.0-L glass beakers at 28°C (Memmert IPP programmable climate cabinet, Germany). This rearing temperature was chosen because it represents the upper limit at which the experimental genotype can survive and reproduce without detriment (it also reflects the upper limit experienced during summer by the population from the experimental genotype was obtained, Kielland *et al.*, 2019, see below). The genotype used in this study had been hatched previously from a resting egg collected in a shallow pond at Værøy Island, northern Norway (67.687°N, 12.672°E). The animals in each beaker were fed *ad libitum* amounts of a commercial shellfish diet (1800, Reed Mariculture Inc., USA) three times per week. Culture medium (ADaM, Klüttgen *et al.*, 1994) in each beaker was changed twice per week when the number of individuals in each beaker was culled to a haphazard selection of 15–20 animals. A 24-h day length mimicked the natural summer photoperiod of the source population (the pond from which the resting egg was collected is located more than 100 km north of the Arctic circle and thus is exposed to continual daylight for several months over summer). The experimental genotype had been acclimated to 28°C under these conditions for a minimum of four asexual generations before measurements of heat tolerance. Each asexual generation was initiated using juveniles from second or later clutches, with each juvenile being sourced from a unique beaker.

### Heat tolerance

We measured heat tolerance as the ability to maintain bodily function, recorded as the ‘time to immobilisation’ (hereafter, *T*_imm_) over temperatures ranging from 35 to 40°C (at increments of 1°C). We estimated *T*_imm_ using an updated version of an R algorithm that objectively identifies the loss of locomotory function from video-derived tracking data (Burton *et al*., 2018). We exposed individual daphnids from two different body size groupings to the exposure temperatures using a watertight thermostatic plate composed of 30 small glass wells. The wells, each open on their upper surface (well diameter 16 mm, depth 21 mm), were inserted in a rectangular 5 × 6 array on a hollow aluminium block (length 265 mm, width 125 mm, thickness 30 mm). One small (mean ± SE = 1.14 mm ± 0.01, range = 0.92–1.51 mm) and one large (mean ± SE = 2.70 mm ± 0.01, size range = 2.37–3.17 mm) individual were selected by eye from each of the experimental cultures (see Fig. S1 for a summary of size data). Body sizes of these animals were recorded from digital images (ImageJ, National Institutes of Health, Bethesda, MD) as the length of the gut (anterior extremity of mid-gut to posterior extremity of the hindgut). The range in sizes measured here encompasses a spectrum of development ranging from recently hatched juveniles through to mature females that had produced multiple clutches of young. Following imaging, animals were briefly returned to the climate cabinet until measurement of heat tolerance. Twenty individuals (10 individuals from each of the 2 size groups) were recorded in each measurement run (one exposure temperature per run and up to two runs per temperature were performed on a given day beginning between 10:30 am and 14:30 pm. Up to three measuring runs were performed at each temperature. Prior to each measuring run, freshly prepared medium (but not aerated) was pipetted into each well, then the plate was heated to the desired exposure temperature by continuously pumping in water from a thermostatically controlled water bath. Within a given measuring run, the 20 individuals were pipetted into the wells (one individual per well), noting the time (in seconds) elapsed from the moment that the first individual was placed in a well (it took between 3–6 min to introduce all 20 individuals to the well plate). The well-plate was filmed from above (Basler aCA1300-60gm, fitted with 5–50-mm, F1.4, CS mount lens) with contrast between the individual in each well and its background obtained by illuminating the well plate from beneath with an LED light board (Huion A4 LED light pad, set to maximum intensity, the lower surface of the well-plate is transparent glass). Video recording began after the last individual within a run was introduced to a well and subsequently ceased when visual inspection indicated that all individuals were motionless. Temperature of medium in several of the empty wells was checked periodically throughout each assay and never deviated by more than 0.1°C from the desired exposure temperature. The resulting video files were processed in Ethovision (version XT 11.5, Noldus Information Technology, The Netherlands, settings: greyscale pixel range 10–145, pixel size range 4–350, sample rate 3 observations s^−1^) to produce a time-series of velocity data for each individual (in mm s^−1^, travelled by the centre-point of each individual). In the modified R algorithm, we calculated *T*_imm_ as the time taken from the introduction of an individual into the well until the time when its swimming velocity was last observed to be above a specified threshold value (see supplementary material for further details of the algorithm). Code for the modified *T*_imm_ algorithm and the tracking data is available at the Dryad Digital Repository (Burton *et al.*, 2020). A full description of the method and hardware can also be found in Burton *et al*. (2018, 2019). In total, we obtained *T*_imm_ estimates for 277 individuals distributed across the 6 exposure temperatures. *T*_imm_ could not be estimated for three individuals due to difficulty in acquiring tracking data.

### Data analysis

We employed linear mixed-effect modelling to describe the influence of body size/age and exposure temperature on *T*_imm_. The full model considered the fixed effects of individual body size, exposure temperature, and an interaction between these on *T*_imm_. Here, the interaction term means that the slope (and thus the temperature sensitivity coefficient, *z*) describing the relationship between exposure temperature and *T*_imm_ can vary between each size grouping. We also fitted two simpler models, one including only the additive effects of body size and exposure temperature (i.e. no effect of body size on z, but potentially on the elevation of the regression, meaning the *y*-intercept for each size grouping can differ), and one including only the effect of exposure temperature. All models were fitted with the same random effect structure, with random intercept terms for beaker (*n* = 10 levels) and measuring run (*n* = 14 levels). We compared the support for these three models using AICc weights (models fit with maximum likelihood). Diagnostics for heteroscedasticity and normality were inspected from residual plots produced from each of the three fitted models. Statistical analysis was conducted in R version 3.5.2 (R Development Core Team, 2019). Our data and R code are available at the Dryad Digital Repository (Burton and Einum, 2020).

## Results and discussion

**Figure 2:**
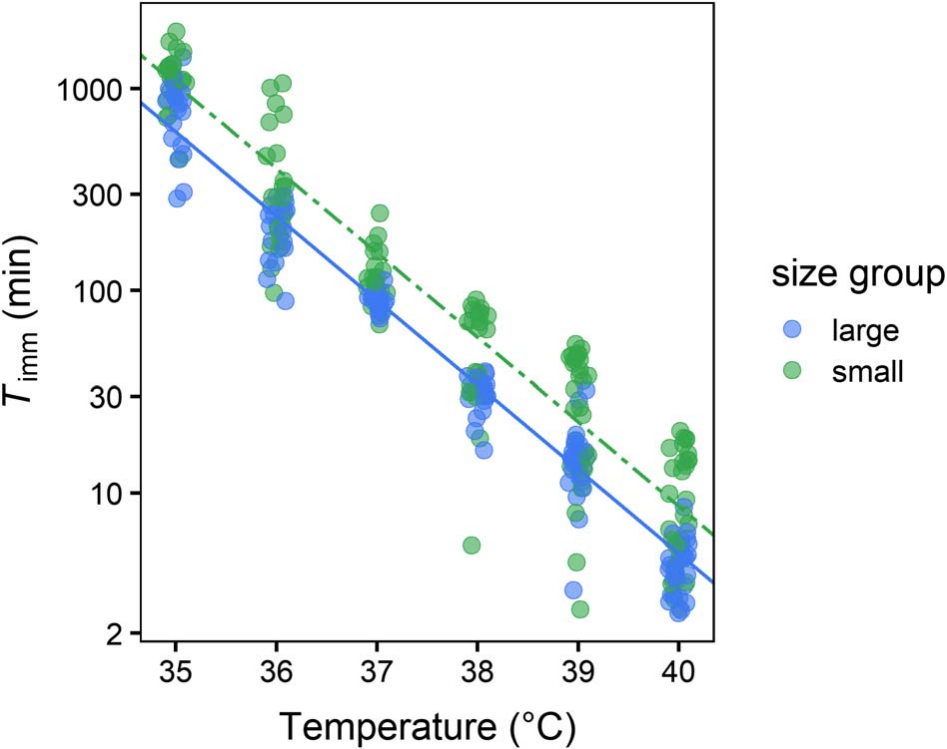
Relationship between time to immobilization, *T*_imm_ (in minutes and exposure temperature for *D. magna* from the small and large size groupings. Solid and dashed lines represent estimates for the additive effect of body size (Model 2). Estimates are plotted for an individual of mean size from the large and small size groupings (mean body size = 2.70 and 1.14 mm, respectively). Estimates for the interactive effect of body size (Model 1) are plotted in the supplementary material. To aid interpretation, a small amount of random noise has been added to each datum point on the *x*-axis.

The two models which considered the effect of body size in addition to that of exposure temperature provided the best fit of the data (Table 1). However, the more complex of these models, which considered the relationship between exposure temperature and body size as an interaction yielded little gain in explanatory power over the simpler model which tested this association additively (i.e. the difference in AICc values between Models 1 & 2 was < 2.0, Table 1). Moreover, inspection of predictions from Models 1 and 2 indicated that the temperature sensitivity of heat tolerance in Daphnia was largely invariant in relation to body size (predictions from the additive model are shown in Fig. 2, whereas predictions for the interactive model are displayed in Fig. S2, parameter estimates from both models are summarised in Table S1). Together, this implies that across all exposure temperatures smaller, younger individuals were able to maintain bodily function for a longer duration than older, larger individuals. Note that more variation in heat tolerance was seemingly evident among individuals within the small size grouping (Fig. 2).

**Table 1:** Candidate models testing the relationship between time to immobilization, *T*_imm_ of *D. magna* in relation to exposure temperature (‘temperature’) and body size (‘size’)

Model	fixed effects	*k*	AIC_c_	ΔAIC_c_	*w_i_*	acc *w_i_*	R^2^
1	temperature × size	7	382.22	0.00	0.67	0.67	0.94
2	temperature + size	6	383.67	1.46	0.33	1.00	0.93
3	temperature	5	463.96	81.74	0.00	1.00	0.91

ΔAIC_c_: difference in AIC_c_ values between a given model and the best fitting model of those considered. *w_i_*: AICc weight or probability that a given model is the best model of those considered. acc *w_i_:* cumulative AICc weight*. R*^2^: conditional *r*-squared for a given model, estimated using the MuMIn library in the R computing environment (Bartoń, 2019)

Using the TDTC framework, which integrates both the intensity and duration of temperature stress to assess heat tolerance, we observed that younger/smaller individuals were better equipped to withstand thermal challenges that are both intense and brief (~5 min) but also more moderate and prolonged (>15 h). The ecological relevance of standard assays of acute temperature tolerance has been questioned on the grounds that organisms are often exposed to measurement temperatures or rates of heating that greatly exceed those encountered in nature (Terblanche *et al.*, 2007; Chown *et al.*, 2009; Rezende *et al.*, 2014). However, consistent with the findings of Jørgensen *et al*. (2019), our results suggest that relationships identified in acute assays of heat tolerance may persist across more moderate temperatures like those implemented here and thus likely reflect the relative heat tolerance of individuals experiencing longer periods (e.g. through a daily cycle) of stressful temperature in nature. Whereas interspecific comparisons of TDTCs have been suggested to demonstrate an evolutionary trade-off between the ability to withstand extreme temperatures and tolerance of moderate exposures (i.e. a high CTmax is seemingly not associated with a high *z* and vice versa, Rezende *et al.*, 2014, but see Jørgensen *et al*., 2019), this does not appear to be the case in the current study. Here, we observed that smaller/younger individuals were more tolerant of the most acute exposures but exhibited similar sensitivity of heat tolerance across measuring temperatures as older/larger individuals. Thus, our results contrast with previous work on a species of inter-tidal snail which employed the TDTC framework, showing that older, larger individuals were more tolerant of relatively high temperature exposures but less resistant to milder exposures of greater duration (Truebano *et al.*, 2018). However, in the latter study, changes in size/age were confounded with distinct developmental changes in morphology and habitat usage. In comparison, juvenile and adult life stages in *D. magna* appear morphologically similar and both inhabit shallow, desiccation prone water bodies (Hanski *et al.*, 1983) where there is likely limited scope for juveniles and adults to exploit different microhabitats. This suggests that differences in heat tolerance among the respective size groupings measured here are unlikely to result from adaptation to different thermal environments.

Whilst we did not attempt to address the precise mechanisms that might underpin the relationship between size/age and heat tolerance reported here, the respiratory protein haemoglobin which facilitates oxygen transport has been proposed as a buffer of high temperature exposure in several groups of aquatic invertebrates (e.g. Seidl *et al.*, 2005). In *D. magna*, production of this protein can decline with age (Green, 1956; Kobayashi, 1982), perhaps as a greater proportion of an individual’s resources are diverted away from growth and maintenance toward investment in eggs (Green, 1956). Hence, further studies on the interactive effects of oxygen availability/delivery and body size on TDTCs may contribute to a greater understanding of the underlying mechanisms. More generally, there is a strong linear relationship between size and age in *D. magna* across the size dichotomy measured here (e.g. Martínez-Jerónimo, 2012), meaning that sexual maturation was the only major developmental transition to occur (all individuals in the large size grouping were sexually mature). Thus, our observation that larger individuals have poorer heat tolerance is unlikely to be explained by greater variation in age among individuals within the large size grouping. However, given the correlation between size and age and the general difficulty in separating the relative effects of the two traits experimentally (for instance, dietary manipulation can slow development but might induce additional physiological variation, e.g. in metabolic rate, Burton *et al.*, 2011, that could also influence heat tolerance), it is unclear to what extent our results might be driven by either trait. Thus the poorer heat tolerance of older, larger individuals might be due to size alone or the shift in allocation of resources associated with maturation (or possibly an interaction between the two).

In summary, by demonstrating that the effects of size on thermal tolerance are largely independent of the intensity and duration of exposure, our results suggest that previous assertions based on the relationship between size/age and acute temperature tolerance may be generalized to milder exposures. Furthermore, in addition to the reductions in size-at-age that have been proposed a developmental response to more gradual warming (Daufresne *et al.*, 2009), our data supports previous assertions that episodes of mortality due to high temperature may actively select against older, larger individuals (Brans *et al.*, 2017, Messmer *et al.*, 2017). Thus, our study adds further empirical support to the hypothesis that changing thermal regimes are contributing to general reductions in body size of aquatic ectotherms (Daufresne *et al.*, 2009; Forster *et al.*, 2012; Evans *et al.*, 2019). Given that body size is often correlated with reproductive output (zooplankton included, Glazier, 1992), the conservation implications of this trend are clear. Older, larger females contribute disproportionally to local populations because they typically produce more, often larger offspring (i.e. so called ‘big old fat fecund females’ or BOFFFs, Hixon *et al.*, 2013; Baudron *et al.*, 2014). Among zooplankton, it is also the same individuals that are the most effective filter feeders (Burns, 1969). Hence, an increase in the frequency of heat-wave-type events likely has the potential to affect the dynamics of zooplankton populations and potentially the wider function of aquatic ecosystems by affecting the individuals that contribute significantly to them.

## Funding

This work was supported by a Marie Skłowdoska-Curie International Fellowship [MSCA-IF 658530] and funding from the Research Council of Norway [Klimaforsk 244046, Centre of Excellence 223257/F50].

## Competing interests

The authors declare no competing or financial interests.

## Supplementary Material

TTL2019_SupplementaryMaterialR1_ConsPhysiol_coaa038
