# Supplementary material for: The old and the large may suffer disproportionately during episodes of high temperature: evidence from a keystone zooplankton species
Source: Conserv Physiol. 2020 Sep 8;8(1):coaa038. doi: 10.1093/conphys/coaa038 (PMC7210711; doi:10.1093/conphys/coaa038)
Supplement: TTL2019_SupplementaryMaterialR1_ConsPhysiol_coaa038 [file ttl2019_supplementarymaterialr1_consphysiol_coaa038.docx]

Supplementary Material

*The old and the large may suffer disproportionately during episodes of high temperature: evidence from a keystone zooplankton species*

Tim Burton^1^* and Sigurd Einum^1^

1. Centre for Biodiversity Dynamics, Department of Biology, Norwegian University of Science and Technology, Realfagbygget, NO-7491 Trondheim, Norway

*corresponding author, email: [tim.burton@ntnu.no](mailto:tim.burton@ntnu.no)

*Size groupings*

**
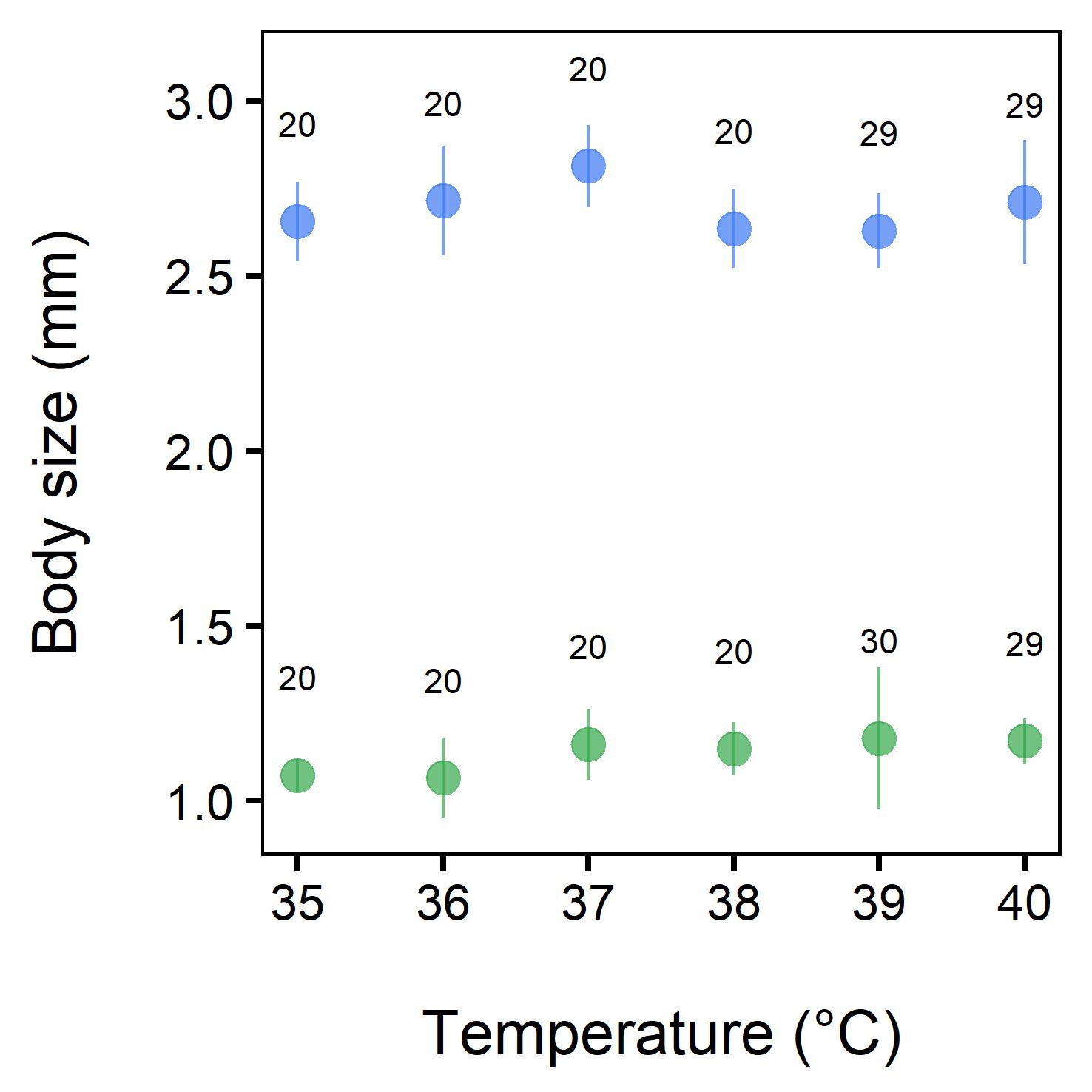
**

**Figure S1.** Mean body sizes (± SD) of *Daphnia magna* from the small and large size groupings that were measured for heat tolerance at each exposure temperature*.* Sample sizes for each size grouping are presented above each datum point.

*T*_imm_ *algorithm*

In the modified algorithm, we calculated *T*_imm_ as the time taken from the introduction of an individual into the well until the time when its swimming velocity was last observed to be above a specified threshold value. To determine this threshold value, we drew upon our observation that tracking software assesses stationary objects as moving to a small degree. To quantify this level of baseline noise, we obtained the maximum recorded velocity of each individual during the final 5 minutes of filming, where manual observation confirmed a complete absence of movement (in the case of the briefest exposures, those performed at 40 °C, this time period was restricted to the final minute). The maximum noise level recorded across all individuals was 0.4 mm s^-1^. When calculating *T*_imm_ we set the threshold swimming velocity in the modified algorithm as twice this maximum noise value (i.e. 0.8 mm s^-1^). Varying this threshold value (by up to ± 50%) had little effect on the resulting parameter estimates and explanatory power of model 2 (Table S1, Fig S3).

**Table S1.** Parameter estimates from models 1 and 2 describing the effect of exposure temperature and body size on heat tolerance, T_imm_, of Daphnia magna.

| term | estimate | SE | t-value | p-value |
| --- | --- | --- | --- | --- |
| *model 1* | | | | |
| intercept | 38.26 | 1.84 | 20.75 | < 0.0001 |
| exposure temperature | -0.89 | 0.05 | -18.22 | < 0.0001 |
| body size | 1.07 | 0.74 | 1.43 | 0.15 |
| exposure temperature × body size | -0.04 | 0.02 | -1.89 | 0.06 |
| *model 2* | | | | |
| intercept | 40.94 | 1.19 | 34.36 | < 0.0001 |
| exposure temperature | -0.96 | 0.03 | -30.55 | < 0.0001 |
| body size | -0.34 | 0.03 | -9.83 | < 0.0001 |

*Supplementary Results*

**
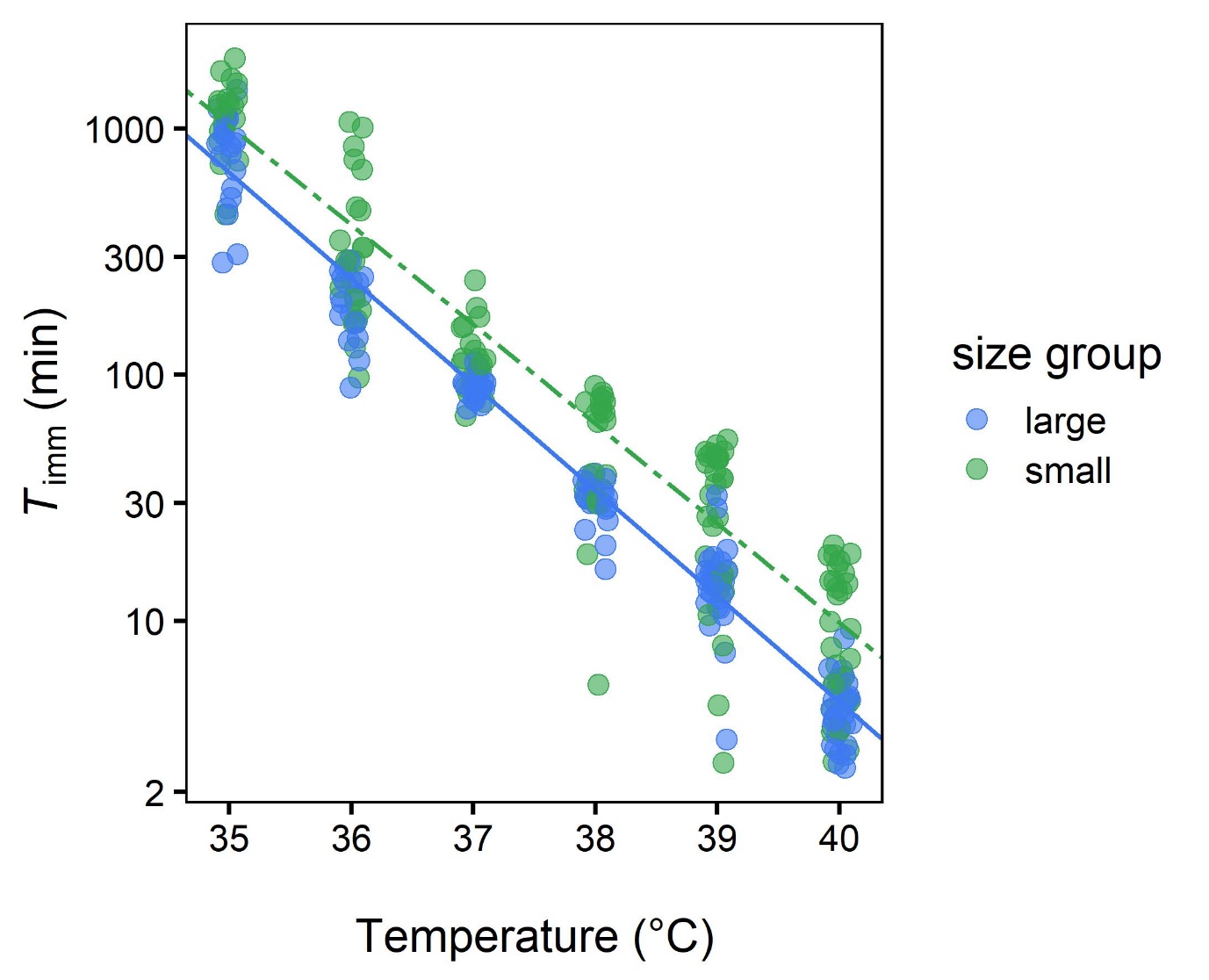
**

**Figure S2.** Relationship between time to immobilization, *T*_imm_ (in minutes, transformed to natural logarithm scale) and exposure temperature for *D. magna* from the small and large size groupings. Solid and dashed lines represent estimates for the interactive effect of body size (model 1, Table S1). Estimates are plotted for an individual of mean size from the large and small size groupings (mean body size = 2.70 and 1.14 mm respectively). Estimates for the additive effect of body size (model 2, Table S1) are plotted in the main text. To aid interpretation, a small amount of random noise has been added to each datum point on the x-axis. Data are available at the Dryad Digital Repository (Burton *et al.*, 2020)


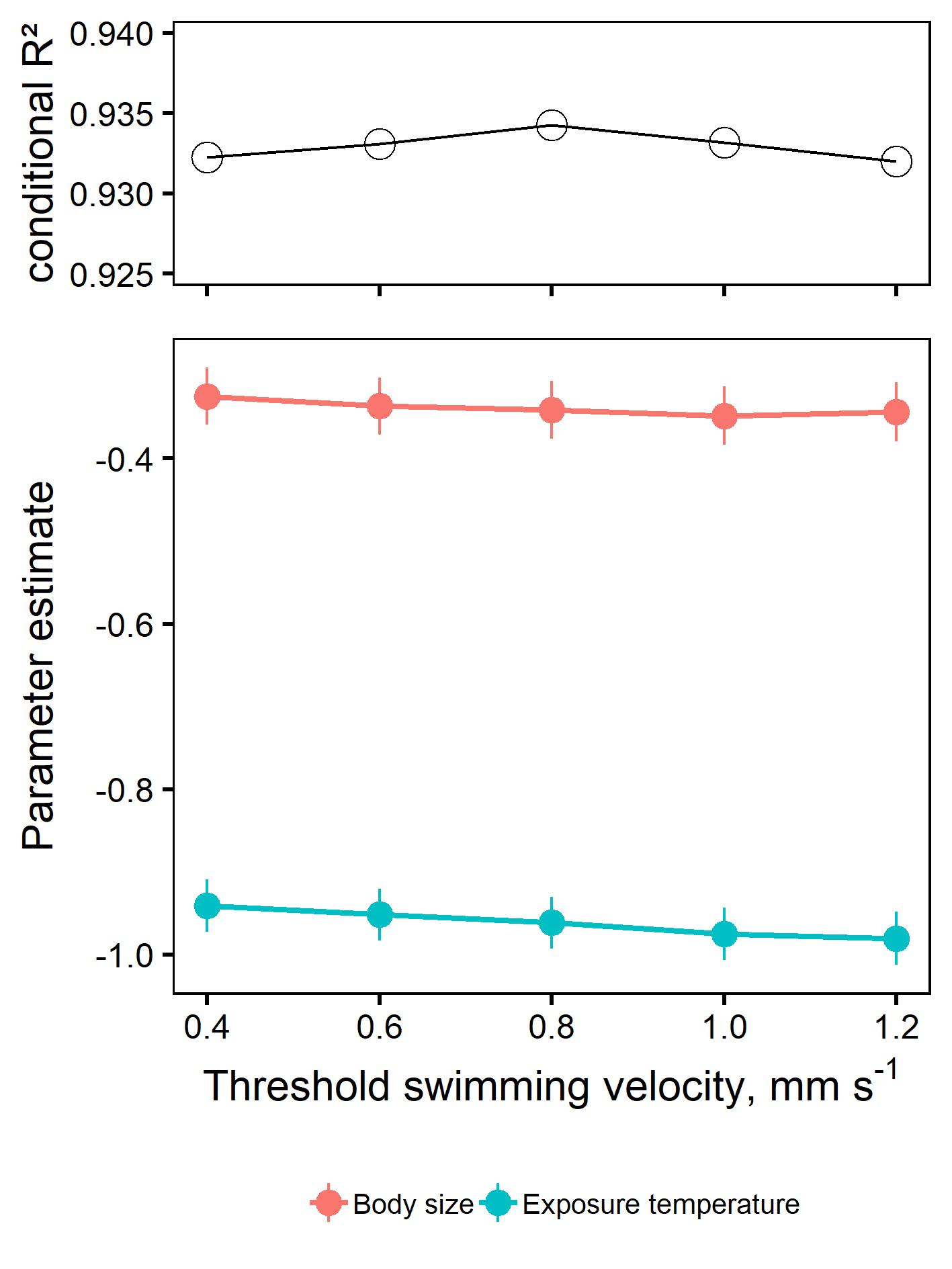


**Figure S3.** Conditional R^2^ values (upper panel) and parameter estimates (± SE, lower panel) for the effect of body size and exposure temperature from five different iterations of model 2 (Table S1) that described an additive relationship between these two variables and *T*_imm_ of *D. magna*. Conditional R^2^ values and parameter estimates are plotted against the different threshold swimming velocities used to calculate the *T*_imm_ data analysed in each iteration of the best-fitting model. *T*_imm_ data presented in the main text was calculated using a threshold value of 0.8 mm s^-1^. The lme4 (Bates *et al.*, 2019) and MuMIn (Bartoń, 2019) libraries in the R computing environment (R Development Core Team, 2019) were used respectively to implement the mixed effect models described here and estimate their conditional R^2^ squared values.

References

**Bartoń K** (2019). Mumin: Multi-model inference, Ed R package version 1.43.6

**Bates D, Maechler M, Bolker B, Walker S, Christensen RHB, Singmann H, Dai B, Scheipl F, Grothendieck G, Green P *et al.*** (2019). Linear mixed-effects models using 'eigen' and s4, Ed R package version 1.1-21

**Burton T, Einum S** (2020) Data from: The old and the large may suffer disproportionately during episodes of high temperature: Evidence from a keystone zooplankton species. Dryad Digital Repository, <https://doi.org/10.5061/dryad.8931zcrmk>

**R Development Core Team** (2019). R: A language and environment for statistical computing, Ed 3.5.3, R Foundation for Statistical Computing, Vienna, Austria
